# Mechanisms in the Catalytic Reduction of N_2_O by CO over the M_13_@Cu_42_ Clusters of Aromatic-like Inorganic and Metal Compounds

**DOI:** 10.3390/molecules28114485

**Published:** 2023-06-01

**Authors:** Ziyang Liu, Haifeng Wang, Yan Gao, Jijun Zhao

**Affiliations:** 1 College of Sciences, Xinjiang Production and Construction Corps Key Laboratory of Advanced Energy Storage Materials and Technology, Shihezi University, Shihezi 832000, China; 20202018011@stu.shzu.edu.cn (Z.L.); whfeng@shzu.edu.cn (H.W.); 2 Key Laboratory of Materials Modification by Laser, Ministry of Education, Ion and Electron Beams, Dalian University of Technology, Dalian 116024, China

**Keywords:** DFT, Cu_55_ cluster, aromatic-like inorganic and metal compounds

## Abstract

Metal aromatic substances play a unique and important role in both experimental and theoretical aspects, and they have made tremendous progress in the past few decades. The new aromaticity system has posed a significant challenge and expansion to the concept of aromaticity. From this perspective, based on spin-polarized density functional theory (DFT) calculations, we systematically investigated the doping effects on the reduction reactions of N_2_O catalyzed by CO for M_13_@Cu_42_ (M = Cu, Co, Ni, Zn, Ru, Rh, Pd, Pt) core–shell clusters from aromatic-like inorganic and metal compounds. It was found that compared with the pure Cu_55_ cluster, the strong M–Cu bonds provide more structural stability for M_13_@Cu_42_ clusters. Electrons that transferred from the M_13_@Cu_42_ to N_2_O promoted the activation and dissociation of the N–O bond. Two possible reaction modes of co-adsorption (L-H) and stepwise adsorption (E-R) mechanisms over M_13_@Cu_42_ clusters were thoroughly discovered. The results showed that the exothermic phenomenon was accompanied with the decomposition process of N_2_O via L-H mechanisms for all of the considered M_13_@Cu_42_ clusters and via E-R mechanisms for most of the M_13_@Cu_42_ clusters. Furthermore, the rate-limiting step of the whole reactions for the M_13_@Cu_42_ clusters were examined as the CO oxidation process. Our numerical calculations suggested that the Ni_13_@Cu_42_ cluster and Co_13_@Cu_42_ clusters exhibited superior potential in the reduction reactions of N_2_O by CO; especially, Ni_13_@Cu_42_ clusters are highly active, with very low free energy barriers of 9.68 kcal/mol under the L-H mechanism. This work demonstrates that the transition metal core encapsulated M_13_@Cu_42_ clusters can present superior catalytic activities towards N_2_O reduction by CO.

## 1. Introduction

Nowadays, air pollution has gradually become a serious environmental problem. Fuel combustion, the emissions of exhaust gases, and excessive fertilization in agriculture have led to a huge production of large amounts of toxic and harmful gases in the atmosphere. Among them, nitrous oxide (N_2_O) and carbon monoxide (CO) have been recognized as two common harmful gases among the emissions of exhaust gases. Specially, N_2_O has been recognized as the main gas that causes the greenhouse effect because it has a global warming potential that is 300 times larger than that of CO_2_ [1,2]. Moreover, it is also a stratospheric ozone depleter [3]. Meanwhile, CO is not only a potential greenhouse gas but can also cause serious harm to human health. In recent years, significant efforts have been devoted to the development and application of the reduction of the two harmful gases [4,5,6,7,8,9,10,11]. One of the most promising methods is to convert them into less harmful N_2_ and CO_2_ gases (N_2_O + CO→N_2_ + CO_2_). This process involves two steps: firstly, a N_2_O molecule reacts to form N_2_ and an adsorbed O atom (N_2_O→N_2_ + O*), then the O* atom reacts with a CO molecule to form CO_2_ (O* + CO→CO_2_) [12]. Notably, the direct reaction of N_2_O with CO molecules shows a high kinetic barrier (about 47.77 kcal/mol) [13], which seriously hinders the reaction’s ability to take place at room temperature. Therefore, it is necessary to develop catalysts that have the properties of being stable, economical, and highly active. Until now, many kinds of catalysts, including noble metal [14], metal zeolites/porphyrins [15], metal alloys, and perovskite-like catalysts [16,17], have been proven to achieve efficient conversions of N_2_O and CO to N_2_ and CO_2_. However, the high cost and difficulties in the mining process (environment, working conditions) have led to a deep search for metal catalysts with similar or improved properties to replace them.

Aromaticity has attracted the most attention in recent years. Li et al. prepared a series of all metal clusters MAl_4_^−^ (M = Li, Na, Cu) and investigated the valence molecular orbitals of Al_4_^2−^ using photoelectron spectroscopy experiments combined with quantum chemical theory calculations [18]. Two *π* electrons occupied completely non-deterministic HOMO orbitals. This study identified MAl_4_^−^ as an aromatic system, which has been confirmed in the scientific research. Zhu et al. reported the first synthesis of metallapentalyne having a transition metal-centered *d* orbital involved in conjugation in osmium metallapentalyne, thus converting the Hückel anti-aromatic nature of metallapentalyne into the Möbius aromatic nature of metallapentalyne [19]. These studies have generated great scientific importance in the extended concept of the aromaticity, which has extended the scope from organic chemistry to metal cluster systems and has opened up a new scientific frontier. In this context, Cu-based aromatic-like metal compound alloys have attracted considerable attentions because of copper’s low cost and excellent properties in various aspects, such as having high stability and long persistence in high-temperature conditions [20,21,22]. In recent years, metallic Cu nanoclusters or Cu-based alloy clusters have been widely applied in various catalytic processes, e.g., hydrogen evolution reaction (HER), NO_x_ reduction, dry reforming of methane (DRM), etc. [23,24,25,26,27]. As for the catalytic reduction of N_2_O by CO reaction, Barabás et al. systematically investigated the performance of Cu_n_ (*n* = 4–15) cluster catalysts and found that Cu_12_ and Cu_14_ clusters were the best catalysts according to the thermodynamic analysis, even at ambient temperatures [28]. Lian et al. systematically studied M@Cu_12_ (M = Cu, Pt, Ru, Pd, Rh) clusters and confirmed that Ru@Cu_12_ and Pt@Cu_12_ clusters exhibited superior catalytic activity via a co-adsorption mechanism [29]. 

As a medium-sized nanocluster (the diameter is about 1 nm) with a typical core–shell structure, the icosahedral Cu_55_ cluster of aromatic-like inorganic and metal compounds is considered to be the global minimum of Cu_55_ clusters [30]. Extensive studies have been carried out to reveal the physical and chemical properties and potentials in the catalysis of this nanocluster material [31,32,33,34,35,36]. Mao et al. reported a DFT-based high-throughput screening method to successfully screen Cu_55-n_M_n_ (M = Co, Ni, Ru, and Rh) core–shell alloy clusters and identified Cu-Ni alloy clusters as a superior electrocatalyst for HER [31]. Cao et al. systematically studied a single Pd-doped Cu_55_ nanoparticle towards propane dehydrogenation and demonstrated that this nanoparticle exhibited superior catalytic activity toward C–H bond activation and significantly reduced side reactions such as deep dehydrogenation [32]. Liu et al. identified a crown jewel-structured Pt_12_Cu_43_ cluster as a promising catalyst candidate for highly efficient and low lost oxygen reduction reaction (ORR) processes [33]. So far, pure or alloyed Cu_55_ nanoclusters have been proven to own great potentials in HER [31], ORR [33], H_2_ dissociation [35], propane dehydrogenation [32], and acetylene selective hydrogenation reactions [36]; however, to the best of our knowledge, no theoretical work has been carried out to investigate their activity towards the selective catalytic reduction of N_2_O via CO (i.e., CO oxidation by N_2_O). We aim to fill up that void with the current work. 

In this paper, by means of DFT calculations, we systematically investigated the structural stability and catalytic performance of pure Cu_55_ core–shell clusters and substituted M_13_@Cu_42_ (M = Co, Ni, Zn, Ru, Rh, Pd, Pt) clusters of aromatic-like inorganic and metal compounds by encapsulating the transition metal atoms as a core in a CO oxidation by N_2_O reaction. The results confirm that the doping of the core metal atoms can greatly affect the structure and electronic and catalytic properties of Cu_55_ clusters. By carefully examining the N_2_O decomposition and CO oxidation processes of these core–shell clusters, we found that Ni_13_@Cu_42_ and Co_13_@Cu_42_ clusters can serve as promising candidates in CO oxidation by N_2_O. The results also reveal that the different reaction mechanisms and the metal modification doping of M_13_@Cu_42_ clusters play a key role in CO oxidation. Our calculations reveal endoplasmically doped, medium-size Cu_55_ clusters that can serve as stable, low-cost, and highly effective catalysts in the selective catalytic reduction of N_2_O by CO.

## 2. Computational Details

All spin-polarized DFT calculations in this paper were performed using the generalized gradient approximation (GGA) of the Perdew–Burke–Ernzerhof (PBE) exchange-correlation functional while adopting the Vienna ab initio simulation package (VASP 5.4.4) software package [37,38,39]. The electron–ion exchange correlation interactions were calculated by using the projector augmented wave (PAW) method. Grimme’s semiempirical DFT-D3 scheme of dispersion correction was implemented to elaborate the van der Waals (vdW) interaction [40]. A plane wave basis was accompanied by a kinetic energy cut-off of 500 eV. The convergence criteria for the structure optimizations were set to 10^−5^ eV, and the Hellmann–Feynman force was less than 0.02 eV Å^−1^. Cluster structures with a vacuum space of 25 Å were applied to ensure negligible interactions within neighboring unit clusters. For the optimized geometries, the K-points was set to be 3 × 3 × 1; they were set to 9 × 9 × 1 for the density of states (DOSs) calculations. The minimum reaction paths for each step of the reaction were considered to be using the climbing image advancement elastic band (CI–NEB) method [41].

In order to characterize the stability of the transition metal (TM) core atoms-substituted M_13_@Cu_42_ clusters, the average binding energy (*E_b_*) was adopted to evaluate the structural stability of core–shell bimetallic clusters, which was calculated according to the following equation:

(1) $$E_{b}=\left(13E_{M}+42E_{Cu}-E_{M_{13}@Cu_{42}}\right)/55$$

where $E_{M_{13}@Cu_{42}}$, *E_Cu,_* and *E_M_* are the total energy of *M_13_@Cu_42_* cluster, energy of the *Cu* atom, and doped metal atom, respectively.

The adsorption energies (*E_ads_*) of the adsorption species on these clusters were defined by the following Equation (2):

(2) $$E_{ads}=E_{total}-E_{species}-E_{cluster},$$

where *E_total_*, *E_specie_*_s_, and *E_cluster_* are the total energies of the total adsorbed systems, isolated adsorption species, and clusters, respectively. A Bader population analysis was adopted to quantify the charge population in each atom in our calculation.

To support the choice of the functional combinations and the computational detail of the basis set described, we provided benchmark calculations of the geometrical parameters for N_2_O, CO, CO_2_, and N_2_. The Cu–Cu average bond length of the Cu_55_ cluster was calculated to be 2.32 Å, which is fairly consistent with the experimental result of 2.37 Å [42]. 

## 3. Results and Discussion

### 3.1. Structure

**Figure 1:**
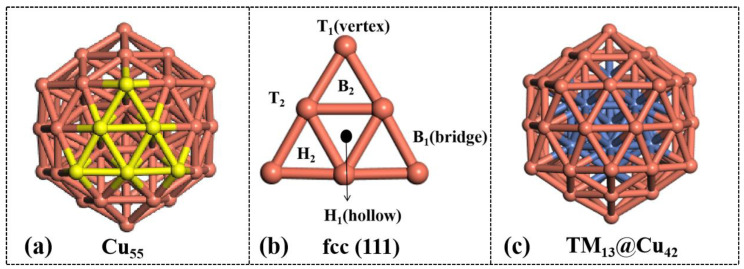
(**a**) Structure of the pristine Cu_55_ cluster; (**b**) atomic arrangement of one of the 20 equivalent facets in the Cu_55_ cluster; (**c**) TM-substituted M_13_@Cu_42_ clusters (TM = Co, Ni, Zn, Ru, Rh, Pd, Pt). The TM and Cu atoms are shown in blue and red, respectively.

The structure of the aromatic-like inorganic and metal compounds Cu_55_ cluster is presented in Figure 1, which had icosahedral (*I_h_)* symmetry and a diameter size of approximately 9.77 Å. The Cu_55_ cluster possessed a multishell structure, in which the outer layer was composed of 42 Cu atoms and the *I_h_* core was formed by 13 copper central atoms. As shown in Figure 1, the surface of Cu_55_ consisted of 20 equivalent triangular fcc (111) facets. The fcc (111) facet in Cu_55_ clusters and aromatic structures are similar. Each facet contained two types of nonequivalent atoms, namely three Cu_1_ atoms at the intersection of five fcc (111) facets (vertex, T) and three Cu_2_ atoms in two contiguous fcc (111) facets (bridge site, B). The different Cu atoms induced different Cu–Cu bond lengths, such as 2.51 Å of d_Cu_1_–Cu_2__ and 2.59 Å of d_Cu_2_–Cu_2__. The distance of the Cu_1_ atoms to the inner layer atom was 4.77 Å with a coordination number of six, while that of the Cu_2_ atoms to the inner layer atom was 4.18 Å with a coordination number of eight. Compared with bulk copper, the different bond lengths in the Cu_55_ cluster endowed its atoms and bonds with higher activity, showing potential in certain catalysis reactions. After the substitution of the core atom, all of the M_13_@Cu_42_ (M = Co, Ni, Zn, Ru, Rh, Pd, Pt) clusters exhibited no obvious structural deformation. 

**Figure 2:**
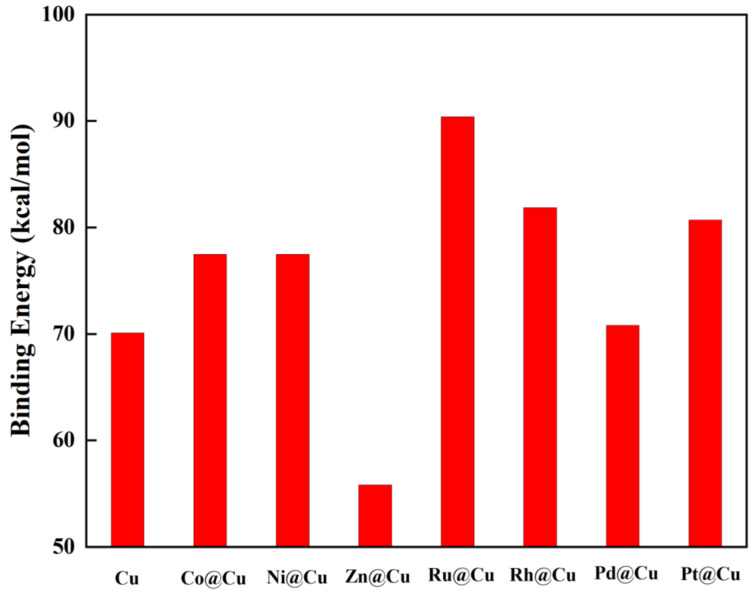
Calculated binding energies of the intrinsic Cu_55_ cluster and M_13_@Cu_42_ (M = Cu, Co, Ni, Zn, Ru, Rh, Pd, Pt)-substituted clusters (absolute value).

Furthermore, the structural stability of the M_13_@Cu_42_ clusters was examined from the binding energy point (Figure 2). The binding energy of the pure Cu_55_ cluster was calculated to be 70.10 kcal/mol, which agrees well with a previous report [43]. The average binding energies of the subsequent clusters were even larger than those of intrinsic Cu_55_ except for Zn_13_@Cu_42_, indicating the strong M–Cu bonds and structural stability of these core-doped clusters, as listed in Table 1. This is reasonable from the viewpoint of the melting points of these metals, e.g., [44]. The energy gap between the highest occupied molecular orbital (HOMO) and the lowest unoccupied molecular orbital (LUMO) of these clusters ranged from 0.01 eV to 0.59 eV. The bond lengths between the M dopants and out-layer Cu atoms ranged from 2.42 Å to 2.65 Å, which was accompanied by an average charge transfer of about 0.01 |e|~0.28 |e| from the TM atoms to the adjacent Cu atoms, as shown in Table 1. Such electron transfers greatly affected the clusters’ catalytic performances, as discussed in the following sections.

### 3.2. Catalytic Properties of M_13_@Cu_42_ Cluster

Before analyzing the CO oxidation by N_2_O, the adsorption of N_2_O on the catalysts was explored. For the neutral state, the N_2_O molecules presented a linear geometry with a N–O bond length of 1.20 Å. Generally, there were two types of geometries for N_2_O adsorption on the catalyst, namely N terminal and O terminal geometries. After geometric optimization, the adsorption energies of N_2_O binding to clusters through the N terminus were smaller than those through the O terminus (about 43.81 kcal/mol). The charge transfer from the system with O terminals to the N_2_O molecule exceeded −0.9|e|; meanwhile, there was no significant charge transfer in the N terminal adsorbed systems. The related parameters and structure of N_2_O adsorption on the Cu_55_ cluster are shown in Table S1 and Figure S1.

Six different adsorption sites on the surface of the pure Cu_55_ cluster were investigated to determine the optimal adsorption configuration for the reaction, including the hollow of the fcc (H_1_, H_2_) facets, top of the Cu_1_ atom (T_1_), top of the Cu_2_ atom (T_2_), bridge of the bond between Cu_1_ and Cu_2_ (B_1_), and bridge of the bond between Cu_2_ and Cu_2_ (B_2_). After geometric optimization with the O terminal, the N_2_O was decomposed to the N_2_ molecule, and the remaining O atom was adsorbed on the Cu_55_ cluster. The average distance of Cu–O was shortened from 2.20 Å to 1.90 Å, while the bond length of N–O was lengthened from 1.20 to 3.19 Å. Clearly, the N–O bond was broken, and the adsorbed O atom prepared for the subsequent oxidation reaction of CO on the cluster. The adsorption energy between the Cu_55_ cluster and N_2_O was obtained as ranging from −48.43 to −53.27 kcal/mol, as shown in Table S1. The adsorption energies of the H_1_ and H_2_ adsorption sites were comparable on the Cu_55_ cluster, but the H_2_ configuration was more easily activated than the H_1_ configuration. The optimization results indicated that the optimized structure of N_2_O adsorption at the B_1_, T_2_, and B_2_ sites was consistent with that located at the H_2_ sites. The subsequent CI–NEB calculation also showed that the remaining O atom was biased to be adsorbed at the H_2_ site after the N_2_ dissociation for the Cu_55_ cluster. The Bader charge analysis proved that approximately 0.96|e| to 0.97|e| was transferred from the clusters to the N_2_O molecule (H_2_ approximately 0.97|e|). Therefore, the H_2_ configuration served as the preferred adsorption site of N_2_O on the Cu_55_ cluster, which is consistent with the literature [45]. For further calculations, the H_2_ site adsorbed to the clusters by the O terminal was selected as the most stable adsorption geometry.

Similarly, the configurations of N_2_O at the H_2_ active site on the doped M_13_@Cu_42_ clusters were studied. The geometry of the N_2_O adsorbs was greatly changed. The atomic average distance of Cu–O was 1.92 Å, while the length of the N–O bond was elongated, ranging from 3.17 Å to 3.58 Å for the M_13_@Cu_42_ clusters. These changes indicated that the N_2_O absorbed by the M_13_@Cu_42_ clusters decomposed to N_2_ and an adsorbed O atom on the M_13_@Cu_42_ cluster for the follow-up reaction. All adsorption energies were negative (−46.12 to −70.33 kcal/mol), suggesting that the adsorption processes for all catalysts were favorable in terms of thermodynamic stability. The Bader charge analysis showed that the M_13_@Cu_42_ clusters transferred 0.97|e| to 1.11|e| charges to N_2_O molecule, which caused its reduction. In other words, the M_13_@Cu_42_ clusters significantly assisted in withdrawing charges from the N_2_O molecule, as indicated in Table 2.

After the analysis of N_2_O adsorption, the entire CO oxidation mechanisms as a result of the N_2_O reaction were investigated by combining the elementary steps associated with all eight kinds of potential catalysts. There were a total of two possible reaction mechanisms, including the E-R and L-H pathways. For the above eight potential catalysts, the most favorable potential energy curves of the CO oxidation as a result of N_2_O processes for all of the possible reaction pathways are presented in Figures S2–S7. The corresponding structures of the reaction intermediates were also explored; the important information is summarized in Table 2.

**Figure 3:**
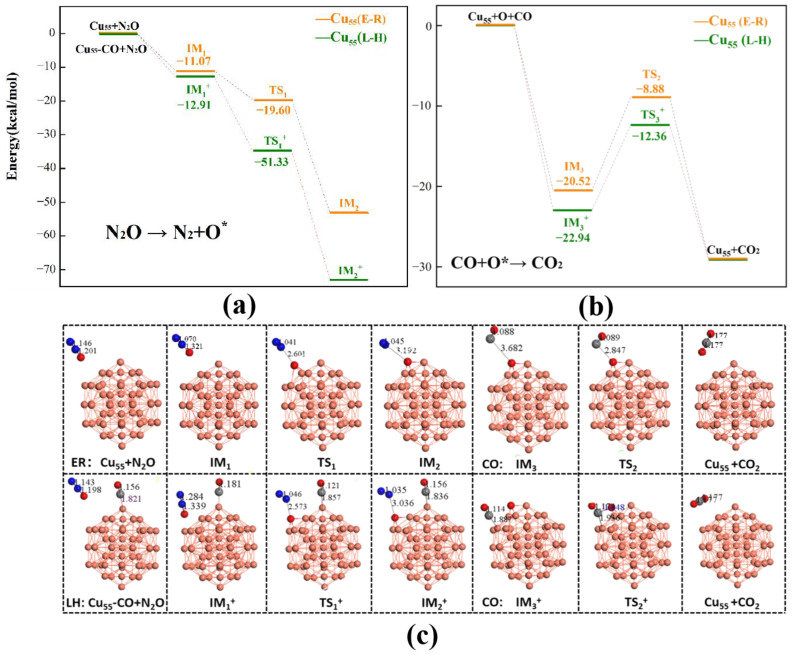
(**a**) N_2_O decomposition to N_2_ and O* and (**b**) CO oxidation by the remaining O* on the Cu_55_ cluster. (**c**) Corresponding optimized intermediates and transition states involved in N_2_O decomposition and CO oxidation on the Cu_55_ cluster (black numbers represent the bond length).

**Figure 4:**
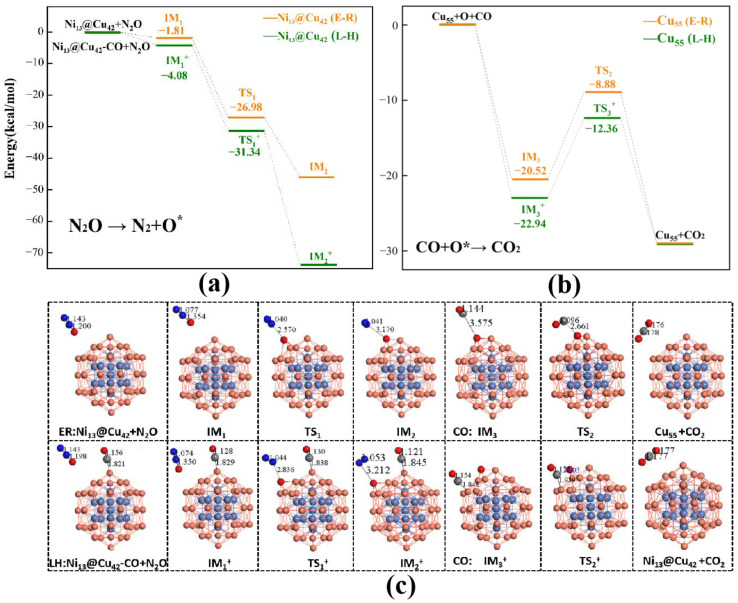
(**a**) N_2_O decomposition to N_2_ and O* and (**b**) CO oxidation by the remaining O* on the Ni_13_@Cu_42_ cluster. (**c**) Corresponding optimized intermediates and transition states involved in N_2_O decomposition and CO oxidation on the Ni_13_@Cu_42_ cluster (black numbers represent the bond length).

Under the L-H mechanism, N_2_O and CO were first co-adsorbed (*N_2_O–*CO) on the catalyst surface, bonding to the underlying two adjacent Cu atoms. Subsequently, the N_2_ was desorbed from the active sites to generate a gaseous N_2_ molecule, and the remaining O atoms were adsorbed on the Cu_55_ cluster. The average distance between *C and Cu was 1.84 Å, and the N–O bond length was 3.04 Å. The adsorption energies of the co-adsorbed CO and N_2_O molecules for the eight types of catalysts were calculated to range from −72.87 to −97.77 kcal/mol. The remaining O atom approached an adsorbed CO molecule and reacted to form CO_2_, which was also exothermic. The adsorption energies of the CO and absorbed O atom ranged from −0.92 to −7.61 kcal/mol. The barrier of the transition state (TS^2+^) for the formation of *O*CO ranged from 9.68 kcal/mol to 19.58 kcal/mol. The transition state involved *O and *CO adsorbates having a distance of 1.93–2.27 Å between them for the M_13_@Cu_42_ clusters. The CO_2_ molecule was directly dissociated from the M_13_@Cu_42_ clusters, indicating the accomplishment of the reaction. By examining the overall CO oxidation as a result of N_2_O processes via the L-H mechanism, the CO oxidation step was considered as the rate-limiting step in the eight catalysts (M_13_@Cu_42_ clusters), which was consistent with the results of the Cu_n_ (*n* = 4–15) clusters [28]. Before the rate-limiting step, the barriers for the N_2_O reduction steps on these eight catalysts were relatively exothermic and barrierless. The released energy ranged from −72.87 to −97.77 kcal/mol for the exothermic reaction of N_2_O reduction. As shown in Figure 3 and Figure 4, the Cu_55_ and Ni_13_@Cu_42_ clusters of catalysts successfully produced N_2_ and CO_2_ with barrier energies of 10.58 and 9.68 kcal/mol, respectively. Notably, the Ni_13_@Cu_42_ cluster possessed the lowest barrier for catalyzing the CO oxidation by the N_2_O reaction.

CO oxidation on the M_13_@Cu_42_ clusters by the E-R mechanism followed similar procedures as those occurring by the L-H mechanism; the difference is whether the CO reactant was physisorbed on the catalyst or not. In the transition state, only one O atom of the reaction intermediates was bound with Cu atoms. As a result, the corresponding barriers were 10.97 kcal/mol and 22.37 kcal/mol, which was higher than those of the L-H mechanism, as shown in Table 2.

**Figure 5:**
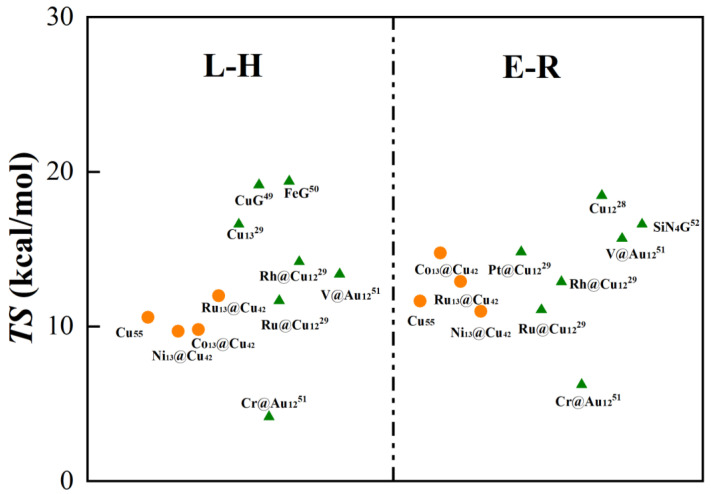
Energy barriers (*TS*) for the CO oxidation via the L-H and E-R mechanisms on Cu_13_/Co_13_/Ni_13_/Ru_13_@Cu_42_ clusters (orange circle) and the barriers for the CO oxidation in some reported studies (green triangle).

Figure 5 summarizes the energy barriers of the rate-limiting step to CO oxidation by the Cu_55_, Co_13_@Cu_42_, Ni_13_@Cu_42_, and Ru_13_@Cu_42_ clusters in this work along with some other reported atomically dispersed catalysts. The barriers of the rate-limiting step to CO oxidation via the L-H mechanism for these four kinds of nanoclusters were obviously lower than that of Cu_13_ (16.60 kcal/mol) [29], Cu-graphene (CuG:19.14 kcal/mol), [46] and Fe-graphene (FeG:19.37 kcal/mol) [47] systems and were comparable with that of a Ru@Cu_12_ (11.66 kcal/mol) [29] catalyst. In addition, the barriers of the rate-limiting step to CO oxidation via the E-R mechanism for these four catalysts were also lower than that of Cu_12_ (18.45 kcal/mol) [28] and SiN_4_G (16.60 kcal/mol) [48], comparable with that of Pt@Cu_12_ (14.81 kcal/mol) [29] and V@Au_12_ (15.68 kcal/mol) [49], and higher than that of Cr@Au_12_ (4.15 kcal/mol or 6.23 kcal/mol) [49], showing great potential in the reaction.

**Figure 6:**
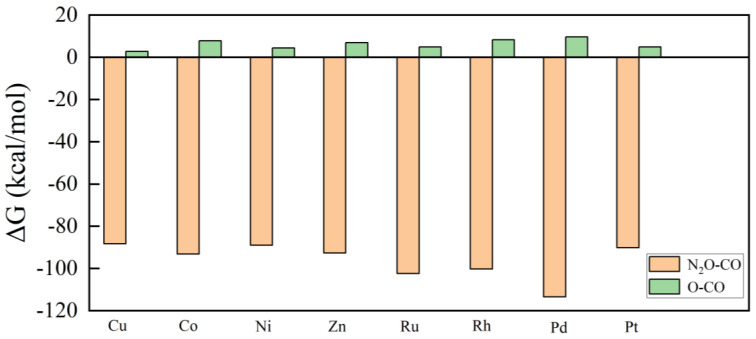
Gibbs free energies (at 1 atm pressure and a temperature of 298.15 K) of the N_2_O reduction by CO reactions via the L-H mechanism for the M_13_@Cu_42_ clusters.

To date, computations refer to hypothetically ideal conditions, where finite temperature and constant pressure are generally desired in experiments. Thus, the Gibbs free energy (Δ*G*) of N_2_O reduction by the CO reaction at 1 atm pressure was calculated and served as a function of a temperature of 298.15 K for each basic procedure; it was computed as:

(3) $$\Delta G=\Delta E+\Delta E_{ZPE}-T\Delta S,$$

where Δ*E* and Δ*E_ZPE_* are the calculated DFT total energy, calculated zero-point energy, and entropic corrections (*T*Δ*S*) at T = 298.15 K. For gas phase molecules, the values of *ZPE* and S came from the NIST database [50]. The Δ*G*, ZPE, and S of the reactant by the thermodynamics systems were calculated (Tables S2 and S3). The reaction occurred readily under ambient conditions in the case of the M_13_@Cu_42_ cluster clusters (except for Pd_13_@Cu_42_), as shown in Figure 6.

The results clearly indicated that Cu_55_ and doped M_13_@Cu_42_ clusters (M = Co, Ni, Zn, Ru, Rh, Pt) enable the reaction process to properly proceed under ambient conditions. Therefore, Cu_55_ and doped M_13_@Cu_42_ clusters are promising catalysts that benefited from the fact that the nitrogen-oxygen bonds were broken without an energy barrier and that the nitrogen molecule readily separated from the cluster. At the same time, these catalysts were able to perform at low temperatures.

The CO oxidation by N_2_O reaction evaluation results showed that doping with different elements of the M_13_@Cu_42_ clusters and different reaction mechanisms had an important effect on the reaction activity. Extensive previous theoretical and experimental works have demonstrated that the rate-limiting step of CO oxidation by the N_2_O reaction depended on the properties of the catalysts. For example, N_2_O reduction possessed a higher energy barrier than CO oxidation on the Ag_7_Au_6_ catalyst [51]. Notably, the potential energy curve analysis indicated that the catalytic activity was tuned by controlling the different active mechanisms. Compared with the E-R mechanism, the bimetallic cluster catalyst under the L-H mechanism reduced the energy barrier of the CO oxidation process. According to the charge transfer in the two mechanisms, more electrons were transferred (approximately 0.20|e|) in the presence of CO, which was favorable compared with the absence of CO. The co-adsorbed CO promoted the conduction of the reaction to some extent. Therefore, the structural robustness and chemical tunability are the prominent advantages of the doped metal cage clusters, making them become a promising family of nanometer catalysts with practical application prospects.

### 3.3. Electronic Structure Analysis

The electronic structure of these bimetallic clusters was deeply analyzed to further understand the catalytic activity of the M_13_@Cu_42_ cluster. The surfaces of all of these clusters exhibited significant charge densities, which corresponded to the electronic states near the Fermi energy level. The electron configuration of N_2_O was ${\left(7\sigma \right)}^{2}{\left(2\pi \right)}^{4}{\left(3\pi \right)}^{0}$, and the frontier molecular orbitals (FMOs) consisted of π–orbitals, where the 2π–HOMO orbital was the bonding orbital of the N–N bond and the antibonding orbital of the N–O bond. 3π–LUMO was the strong antibonding orbital between all atoms [52].

There was a small charge transfer from the N_2_O molecule to the metallic clusters when the N_2_O molecule was attached to the surface of clusters through its O end. When N_2_O was adsorbed, a sizeable electron density rearrangement appeared on the shell atoms; the core atoms were not significantly affected. In the alloy metal clusters, the electrons on the shell Cu atoms were transferred to the core dopant atom. Due to the reduction of electrons in the shell Cu atoms, the electrophilicity of the Cu atoms was enhanced, which makes it easier for alloy metal clusters to adsorb N_2_O compared with pure Cu_55_ clusters. The adsorbability of the M_13_Cu_42_ cluster was better than that of the Cu_55_ cluster in the presence of greater electron transfer, which was consistent with the adsorption energy analysis. CO oxidation served as the rate-limiting step during the reaction, and a cluster complex with residual O was produced in both mechanisms; therefore, the interaction between O and CO played an important role. The CO molecule was attached to the metallic clusters through the C atom, and the electron of the CO molecule was transferred to the metallic clusters. For example, the number of charge transfers from CO to the bimetallic clusters was larger than that from CO to the pure Cu_55_ cluster, which led to a higher catalytic performance for CO oxidation on the alloy clusters.

**Figure 7:**
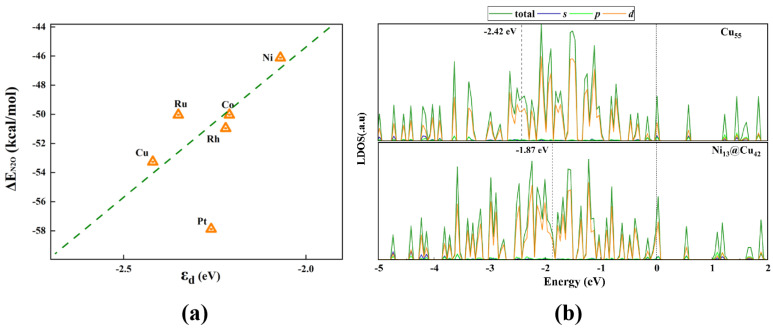
(**a**) *d* orbital centers as a function of ΔE_N2O_ for different M_13_@Cu_42_ clusters. (**b**) Local density of states (LDOSs) of Ni-doped Ni_13_@Cu_42_ and Cu_55_ clusters. The black dashed lines and numbers next to them indicate the *d* orbital centers of each system.

All of these clusters show prominent charge densities on the Cu cage surface, which correspond to the electronic states near the Fermi level and are responsible for the chemical reactivity. As shown in Figure 7a, the M_13_@Cu_42_ cluster with a lower *d* orbital center provided a stronger adsorption strength with the CO molecule. Such a trend of activity was also observed in previous studies [49,53]. This interaction makes it easier for N_2_O to obtain electrons, meaning that the N–O bond of N_2_O is more easy to break. Intuitively, less charge transfer between M–Cu indicates a weaker bonding between M and Cu atoms and an enhanced unsaturation of the Cu outer cage, resulting in a higher reactivity of the surface Cu atoms to CO. The activity of the M_13_@Cu_42_ clusters can be further associated with the *d* orbital center of the cluster, defined as [54]:

(4) $$\varepsilon_{d}=\frac{\int_{-\infty }^{0}ED\left(E\right)dE}{\int_{-\infty }^{0}D\left(E\right)dE},$$

where *D*€ is the local density of states (LDOSs) of the *d* orbitals of the cluster at a given energy E; the integral is taken from all occupied states, and the highest occupied molecular orbital (HOMO) is set to zero. The LDOSs of the M_13_@Cu_42_ clusters are shown in Figure 7b and Figure S8.

According to the picture of the extended Hückel theory [55], a deeper *d* orbital level of the catalyst leads to a lower hopping matrix element and stronger binding strength with the adsorbate. Therefore, the binding ability and activity of the M_13_@Cu_42_ clusters are related to the *d* orbital centers of the clusters, and the catalytic performance can be optimized by selecting suitable doping elements and even by designing ideal catalysts for various reactions.

## 4. Conclusions

In conclusion, M_13_@Cu_42_ (M = Cu, Co, Ni, Zn, Ru, Rh, Pd, Pt) core–shell clusters of aromatic-like inorganic and metal compounds, where transition metal atoms acted as the core for the selective catalytic reduction of N_2_O via CO, were systematically investigated using periodic spin-polarized first-principles calculations. The results show that the stability of these M_13_@Cu_42_ clusters are significantly higher than that of the intrinsic Cu_55_ cluster. Meanwhile, the total charge transfers from the shell to the central doped atoms were shown to increase. The doping of the central metal atom affected the catalytic and electronic properties of the clusters. A portion of the M_13_@Cu_42_ clusters that had suitable binding capacities comprised a low potential barrier. Especially, the kinetic barrier of Ni_13_@Cu_42_ was 9.68 kcal/mol for CO oxidation under the L-H mechanism. The L-H mechanism, which stemmed from gas molecule co-adsorption on the M_13_@Cu_42_ clusters and transition metal modification doping, played a key role in the adsorption of CO oxidation. Our calculations revealed the use of endoplasmically doped copper clusters as a novel stable subnanocatalyst, which is a promising material for high-performance catalytic media.

## Figures and Tables

**Table 1:** Structural, energetic, and electronic properties of M_13_@Cu_42_ clusters, including the average M–M and M–Cu bond lengths (d_Cu–Cu_, d_M–Cu_), binding energies (E_b_), magnetic moment (Mag), average charges transferred from M to Cu atoms (CT_M_), average charges transferred from the shell-Cu atoms to the core-M metal atoms (CT_Cu_), *d* orbital centers (ε_d_), and HOMO–LUMO gaps (Eg).

M	d_Cu-M_ (Å)	d_M-M_ (Å)	E_b_ (kcal/mol)	Mag (μB)	CT_M_ (e)	CT_Cu_ (e)	ε_d_ (eV)	E_g_ (eV)
Cu	2.42	2.49	−70.10	3	+0.06	−0.017	2.42	0.1
Co	2.42	2.45	−77.48	21	−0.05	0.017	2.21	0.01
Ni	2.43	2.45	−77.48	8	−0.01	0.003	2.07	0.01
Zn	2.44	2.59	−55.81	2	−0.16	0.052	3.51	0.59
Ru	2.62	2.61	−90.40	4	+0.01	−0.002	2.45	0.02
Rh	2.48	2.69	−81.86	9	+0.12	−0.035	2.22	0.02
Pd	2.48	2.70	−70.79	2	+0.18	−0.054	2.11	0.17
Pt	2.65	2.82	−80.71	0	+0.28	−0.086	2.26	0.16

**Table 2:** Adsorption energies of N_2_O and CO molecules on M_13_@Cu_42_ clusters (ΔE_N2O_, ΔE_CO_), adsorption energies of co-adsorbed N_2_O and CO molecules on M_13_@Cu_42_ clusters (ΔE_N2O-CO_, ΔE_O-CO_), charge transfer between the molecule and cluster (CT), and energy barriers of the CO oxidation (TS).

	E-R				L-H			
M_13_@Cu_42_	ΔE_N2O_ (kcal/mol)	ΔE_CO_ (kcal/mol)	CT (e)	*TS* (kcal/mol)	ΔE_N2O-CO_ (kcal/mol)	ΔE_O-CO_ (kcal/mol)	CT (e)	*TS* (kcal/mol)
Cu	−53.27	−27.44	0.97	11.64	−72.87	−7.61	1.09	10.58
Co	−50.04	−28.59	1.07	14.75	−77.48	−2.77	1.10	9.80
Ni	−46.12	−32.51	1.08	10.97	−74.02	−5.53	1.08	9.68
Zn	−50.27	−28.36	0.97	17.35	−78.40	−2.31	1.09	19.58
Ru	−50.04	−28.36	1.11	12.90	−86.71	−5.77	1.12	11.99
Rh	−50.96	−27.67	1.10	13.99	−84.40	−2.54	1.13	12.53
Pd	−70.33	−20.06	1.09	22.37	−97.77	−0.92	1.18	18.22
Pt	−57.88	−20.75	1.11	14.93	−75.41	−4.84	1.12	12.47

## Data Availability

The data that support the findings of this study are available within the article and its Supplementary Materials.

## Supplementary Materials

The following supporting information can be downloaded at: https://www.mdpi.com/article/10.3390/molecules28114485/s1.

## References

[1] Trogler W.C. (1999). Physical properties and mechanisms of formation of nitrous oxide. Coord. Chem. Rev..

[2] Ravishankara A., Daniel J.S., Portmann R.W. (2009). Nitrous oxide (N_2_O): The dominant ozone-depleting substance emitted in the 21st century. Science.

[3] Dameris M. (2010). Depletion of the ozone layer in the 21st century. Angew. Chem. Int. Ed..

[4] Esrafili M.D., Janebi H., Mousavian P. (2021). Single Al atom anchored on defective MoS2: An efficient catalytic site for reduction of greenhouse N_2_O gas by CO or C_2_H_4_ molecules. Appl. Surf. Sci..

[5] Shao L., Chen J., Wang K., Mei J., Tan T., Wang G., Liu K., Gao X. (2022). Highly precise measurement of atmospheric N2O and CO using improved White cell and RF current perturbation. Sens. Actuators B Chem..

[6] Esrafili M.D., Nejadebrahimi B. (2019). N_2_O reduction over a porous Si-decorated carbon nitride fullerene: A DFT study. Chem. Phys. Lett..

[7] Sittiwong J., Jaturajamrenchai T., Wongkampuan P., Somwatcharajit N., Impeng S., Maihom T., Probst M., Limtrakul J. (2021). Modulating the catalytic activity of metal-organic frameworks for CO oxidation with N_2_O through an oriented external electric field. Mol. Catal..

[8] Amaya-Roncancio S., Reinaudi L., Gimenez M.C. (2020). Adsorption and dissociation of CO on metal clusters. Mater. Today Commun..

[9] Chen P., Gu M., Chen G., Liu F., Lin Y. (2019). DFT study on the reaction mechanism of N_2_O reduction with CO catalyzed by char. Fuel.

[10] Khan A.A., Ahmad R., Ahmad I. (2021). Removal of nitrous and carbon mono oxide from flue gases by Si-coordinated nitrogen doped C60-fullerene: A DFT approach. Mol. Catal..

[11] You Y., Chen S., Li J., Zeng J., Chang H., Ma L., Li J. (2020). Low-temperature selective catalytic reduction of N_2_O by CO over Fe-ZSM-5 catalysts in the presence of O_2_. J. Hazard. Mater..

[12] Boehme D.K., Schwarz H. (2005). Gas-phase catalysis by atomic and cluster metal ions: The ultimate single-site catalysts. Angew. Chem. Int. Ed..

[13] Delabie A., Vinckier C., Flock M., Pierloot K. (2001). Evaluating the activation barriers for transition metal N_2_O reactions. J. Phys. Chem. A.

[14] Campa M.C., Doyle A.M., Fierro G., Pietrogiacomi D. (2022). Simultaneous abatement of NO and N_2_O with CH_4_ over modified Al_2_O_3_ supported Pt, Pd, Rh. Catal. Today.

[15] Okemoto A., Harada M.R., Ishizaka T., Hiyoshi N., Sato K. (2020). Catalytic performance of MoO_3_/FAU zeolite catalysts modified by Cu for reverse water gas shift reaction. Appl. Catal. A Gen..

[16] Kim K., Baek S., Kim J.J., Han J.W. (2020). Catalytic decomposition of N_2_O on PdxCuy alloy catalysts: A density functional theory study. Appl. Surf. Sci..

[17] Richards N., Carter J.H., Parker L.A., Pattisson S., Hewes D.G., Morgan D.J., Davies T.E., Dummer N.F., Golunski S., Hutchings G.J. (2020). Lowering the operating temperature of perovskite catalysts for N_2_O decomposition through control of preparation methods. ACS Catal..

[18] Li X., Kuznetsov A.E., Zhang H.-F., Boldyrev A.I., Wang L.-S. (2001). Observation of all-metal aromatic molecules. Science.

[19] Zhu C., Li S., Luo M., Zhou X., Niu Y., Lin M., Zhu J., Cao Z., Lu X., Wen T. (2013). Stabilization of anti-aromatic and strained five-membered rings with a transition metal. Nat. Chem..

[20] Niu T., Liu G., Chen Y., Yang J., Wu J., Cao Y., Liu Y. (2016). Hydrothermal synthesis of graphene-LaFeO_3_ composite supported with Cu-Co nanocatalyst for higher alcohol synthesis from syngas. Appl. Surf. Sci..

[21] Wang X., Qiu S., Feng J., Tong Y., Zhou F., Li Q., Song L., Chen S., Wu K.H., Su P. (2020). Confined Fe–Cu clusters as sub-nanometer reactors for efficiently regulating the electrochemical nitrogen reduction reaction. Adv. Mater..

[22] Nicholas K.M., Lander C., Shao Y. (2022). Computational Evaluation of Potential Molecular Catalysts for Nitrous Oxide Decomposition. Inorg. Chem..

[23] Liu Y., Huang L., Zhu X., Fang Y., Dong S. (2020). Coupling Cu with Au for enhanced electrocatalytic activity of nitrogen reduction reaction. Nanoscale.

[24] Kim C., Kim J. (2022). Comparative evaluation of artificial neural networks for the performance prediction of Pt-based catalysts in water gas shift reaction. Int. J. Energy Res..

[25] Ko B.H., Hasa B., Shin H., Jeng E., Overa S., Chen W., Jiao F. (2020). The impact of nitrogen oxides on electrochemical carbon dioxide reduction. Nat. Commun..

[26] Pan K.L., Young C.W., Pan G.T., Chang M.B. (2020). Catalytic reduction of NO by CO with Cu-based and Mn-based catalysts. Catal. Today.

[27] Andana T., Rappé K.G., Nelson N.C., Gao F., Wang Y. (2022). Selective catalytic reduction of NOx with NH_3_ over Ce-Mn oxide and Cu-SSZ-13 composite catalysts–Low temperature enhancement. Appl. Catal. B Environ..

[28] Barabás J.L., Höltzl T. (2016). Reaction of N_2_O and CO catalyzed with small copper clusters: Mechanism and design. J. Phys. Chem. A.

[29] Lian X., Guo W., He B., Yu B., Chen S., Qin D., Chen F. (2020). Insights of the mechanisms for CO oxidation by N_2_O over M@Cu_12_ (M = Cu, Pt, Ru, Pd, Rh) core-shell clusters. Mol. Catal..

[30] Jiang T., Mowbray D., Dobrin S., Falsig H., Hvolbæk B., Bligaard T., Nørskov J.K. (2009). Trends in CO oxidation rates for metal nanoparticles and close-packed, stepped, and kinked surfaces. J. Phys. Chem. C.

[31] Mao X., Wang L., Xu Y., Wang P., Li Y., Zhao J. (2021). Computational high-throughput screening of alloy nanoclusters for electrocatalytic hydrogen evolution. npj Comput. Mater..

[32] Cao X., Ji Y., Luo Y. (2015). Dehydrogenation of propane to propylene by a Pd/Cu single-atom catalyst: Insight from first-principles calculations. J. Phys. Chem. C.

[33] Liu Q., Wang X., Li L., Song K., Qian P., Feng Y.P. (2022). Design of platinum single-atom doped metal nanoclusters as efficient oxygen reduction electrocatalysts by coupling electronic descriptor. Nano Res..

[34] Liu C., Dong H., Ji Y., Rujisamphan N., Li Y. (2019). High-performance hydrogen evolution reaction catalysis achieved by small core-shell copper nanoparticles. J. Colloid Interface Sci..

[35] Zhang R., Wang Y., Wang B., Ling L. (2019). Probing into the effects of cluster size and Pd ensemble as active center on the activity of H2 dissociation over the noble metal Pd-doped Cu bimetallic clusters. Mol. Catal..

[36] Zhao B., Zhang R., Huang Z., Wang B. (2017). Effect of the size of Cu clusters on selectivity and activity of acetylene selective hydrogenation. Appl. Catal. A Gen..

[37] Kresse G., Furthmüller J. (1996). Efficient iterative schemes for ab initio total-energy calculations using a plane-wave basis set. Phys. Rev. B.

[38] Kresse G., Joubert D. (1999). From ultrasoft pseudopotentials to the projector augmented-wave method. Phys. Rev. B.

[39] Perdew J.P., Burke K., Ernzerhof M. (1996). Generalized gradient approximation made simple. Phys. Rev. Lett..

[40] Grimme S. (2006). Semiempirical GGA-type density functional constructed with a long-range dispersion correction. J. Comput. Chem..

[41] Henkelman G., Uberuaga B.P., Jónsson H. (2000). A climbing image nudged elastic band method for finding saddle points and minimum energy paths. J. Chem. Phys..

[42] Tang D., Chen Z., Hu J., Sun G., Lu S., Hu C. (2012). CO oxidation catalyzed by silver nanoclusters: Mechanism and effects of charge. Phys. Chem. Chem. Phys..

[43] Austin N., Butina B., Mpourmpakis G. (2016). CO_2_ activation on bimetallic CuNi nanoparticles. Prog. Nat. Sci. Mater. Int..

[44] McEuen P., Kittel C. (2005). Introduction to Solid State Physics.

[45] Tang D., Zhang J. (2013). Theoretical investigation on CO oxidation catalyzed by a copper nanocluster. RSC Adv..

[46] Akça A., Karaman O., Karaman C. (2021). Mechanistic insights into catalytic reduction of N_2_O by CO over Cu-embedded graphene: A density functional theory perspective. ECS J. Solid State Sci. Technol..

[47] Wannakao S., Nongnual T., Khongpracha P., Maihom T., Limtrakul J. (2012). Reaction mechanisms for CO catalytic oxidation by N_2_O on Fe-embedded graphene. J. Phys. Chem. C.

[48] Junkaew A., Namuangruk S., Maitarad P., Ehara M. (2018). Silicon-coordinated nitrogen-doped graphene as a promising metal-free catalyst for N_2_O reduction by CO: A theoretical study. RSC Adv..

[49] Zhou S., Pei W., Du Q., Zhao J. (2019). Foreign atom encapsulated Au 12 golden cages for catalysis of CO oxidation. Phys. Chem. Chem. Phys..

[50] Chase M.W. (1996). NIST–JANAF thermochemical tables for the bromine oxides. J. Phys. Chem. Ref. Data.

[51] Wongnongwa Y., Namuangruk S., Kungwan N., Jungsuttiwong S. (2017). Mechanistic study of CO oxidation by N_2_O over Ag_7_Au_6_ cluster investigated by DFT methods. Appl. Catal. A Gen..

[52] Piskorz W., Zasada F., Stelmachowski P., Kotarba A., Sojka Z. (2013). DFT modeling of reaction mechanism and ab initio microkinetics of catalytic N_2_O decomposition over alkaline earth oxides: From molecular orbital picture account to simulation of transient and stationary rate profiles. J. Phys. Chem. C.

[53] Pei W., Zhou S., Bai Y., Zhao J. (2018). N-doped graphitic carbon materials hybridized with transition metals (compounds) for hydrogen evolution reaction: Understanding the synergistic effect from atomistic level. Carbon.

[54] Zhou S., Yang X., Pei W., Liu N., Zhao J. (2018). Heterostructures of MXenes and N-doped graphene as highly active bifunctional electrocatalysts. Nanoscale.

[55] Xin H., Linic S. (2010). Communications: Exceptions to the d-band model of chemisorption on metal surfaces: The dominant role of repulsion between adsorbate states and metal d-states. J. Chem. Phys..

