# Supplementary material for: Mechanisms in the Catalytic Reduction of N2O by CO over the M13@Cu42 Clusters of Aromatic-like Inorganic and Metal Compounds
Source: Molecules. 2023 Jun 1;28(11):4485. doi: 10.3390/molecules28114485 (PMC10254853; doi:10.3390/molecules28114485)
Supplement: Supplementary file 1 [file molecules-28-04485-s001.zip › molecules-2403720-supplementary.pdf]

Support Information

Mechanisms in the Catalytic Reduction of N_2O by CO over the $M_{13}@Cu_{42}$ Clusters of Aromatic-Like Inorganic and Metal Compounds

Table of Contents

Figure S1. The initial and final structures of N_2O adsorption on Cu_{55} cluster systems.
Figure S2. Potential energy profiles, the optimized intermediates and transition states for N_2O decomposition to N_2 on the $Co_{13}@Cu_{42}$ cluster.
Figure S3. Potential energy profiles, the optimized intermediates and transition states for N_2O decomposition to N_2 on the $Zn_{13}@Cu_{42}$ cluster.
Figure S4. Potential energy profiles, the optimized intermediates and transition states for N_2O decomposition to N_2 on the $Ru_{13}@Cu_{42}$ cluster.
Figure S5. Potential energy profiles, the optimized intermediates and transition states for N_2O decomposition to N_2 on the $Rh_{13}@Cu_{42}$ cluster.
Figure S6. Potential energy profiles, the optimized intermediates and transition states for N_2O decomposition to N_2 on the $Pd_{13}@Cu_{42}$ cluster.
Figure S7. Potential energy profiles, the optimized intermediates and transition states for N_2O decomposition to N_2 on the $Pt_{13}@Cu_{42}$ cluster.
Figure S8. Local density of states (LDOS) of $M_{13}@Cu_{42}$ clusters (M = Cu, Co, Ni, Zn, Ru, Rh, Pd, Pt).
Table S1. Adsorption energies of different adsorption modes (N, O) and different adsorption sites for N_2O on Cu_{55} cluster (ΔE_{ads}), charge transfer between the molecule and cluster (CT).
Table S2. The molecular energies, Zero-point energy (ZPE), entropic corrections (TΔS) at T = 298.15 K to the free energies and (ZPE−TΔS) values of the gas-phase.
Table S3. The free energies of the adsorbed species at T = 298.15 K.

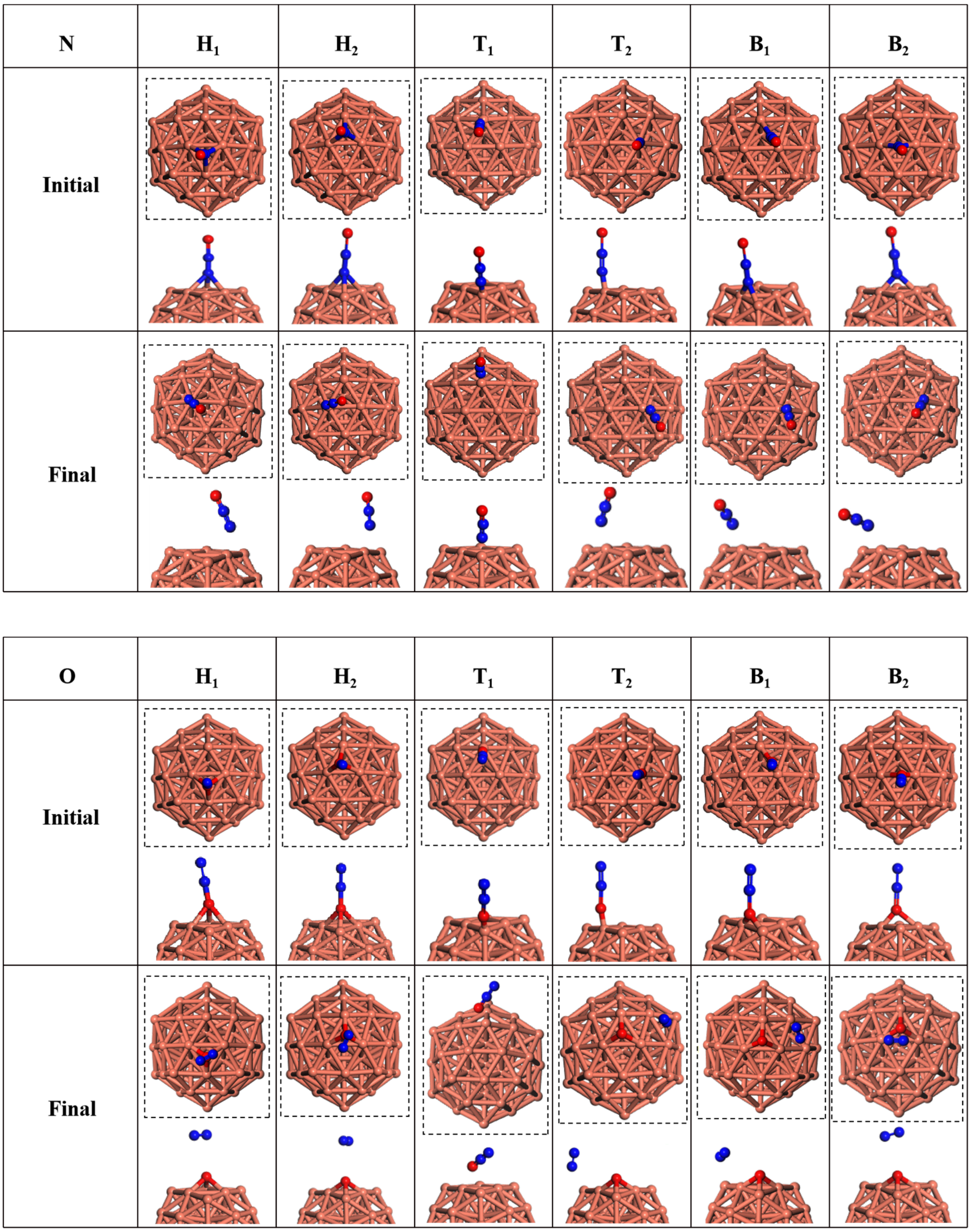

Figure S1. The initial and final structures of N_2O adsorption on Cu_{55} cluster systems. The left superscript "N" or "O" indicates the N terminal or O terminal manners in N_2O adsorption.

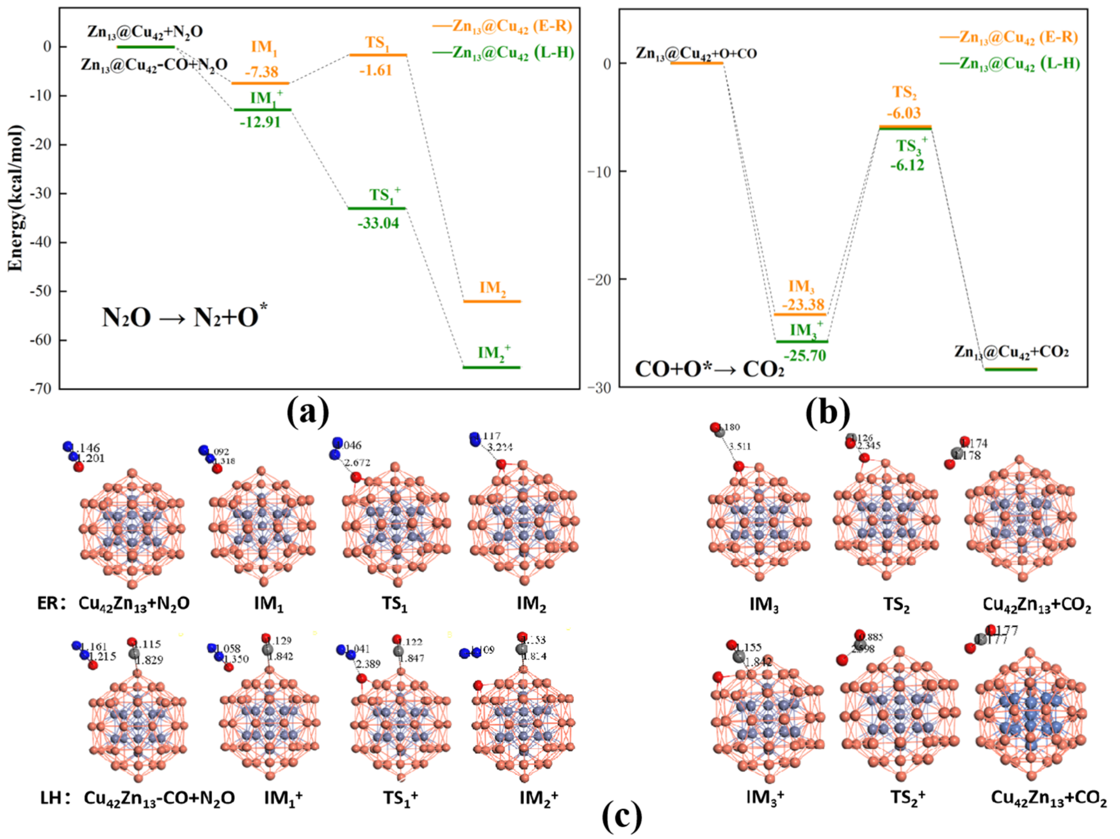

Figure S2. (a) and (b) Potential energy profiles for N_2O decomposition to N_2 on the $Co_{13}@Cu_{42}$ cluster. (c) Corresponding optimized intermediates and transition states involved in CO oxidation on the $Co_{13}@Cu_{42}$ cluster.

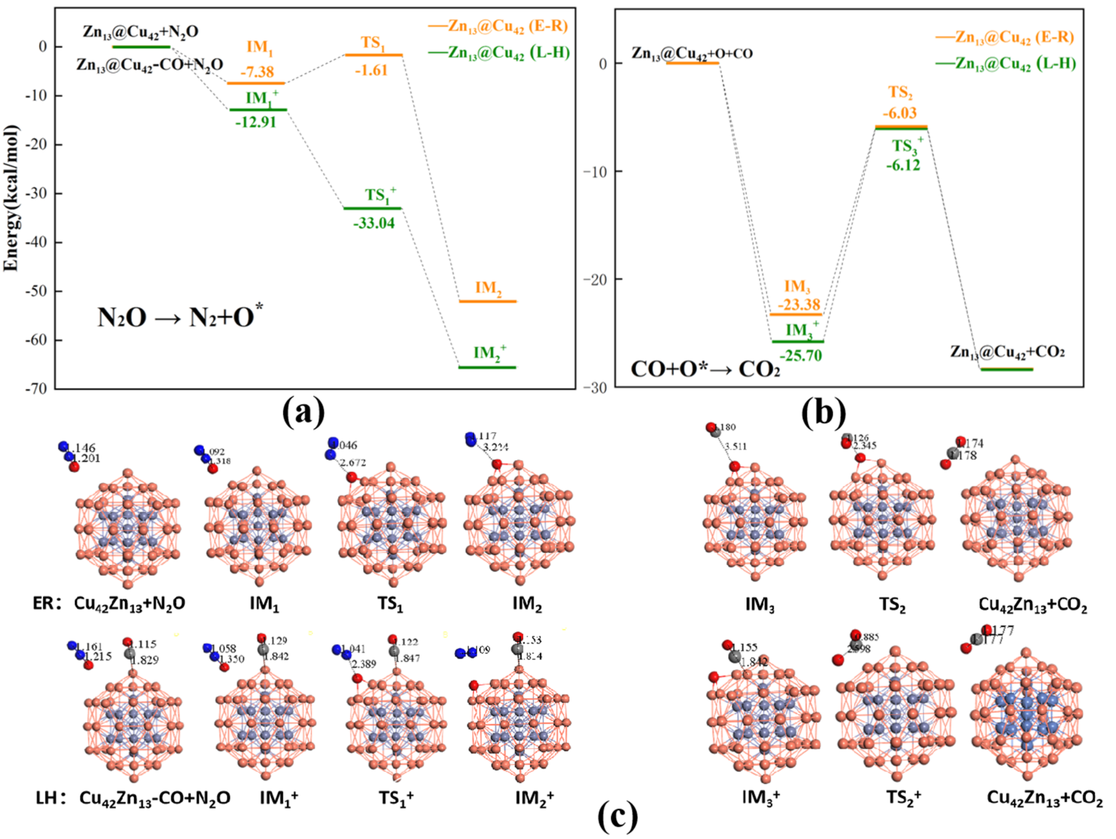

Figure S3. (a) and (b) Potential energy profiles for N_2O decomposition to N_2 on the $Zn_{13}@Cu_{42}$ cluster. (c) Corresponding optimized intermediates and transition states involved in CO oxidation on the $Zn_{13}@Cu_{42}$ cluster.

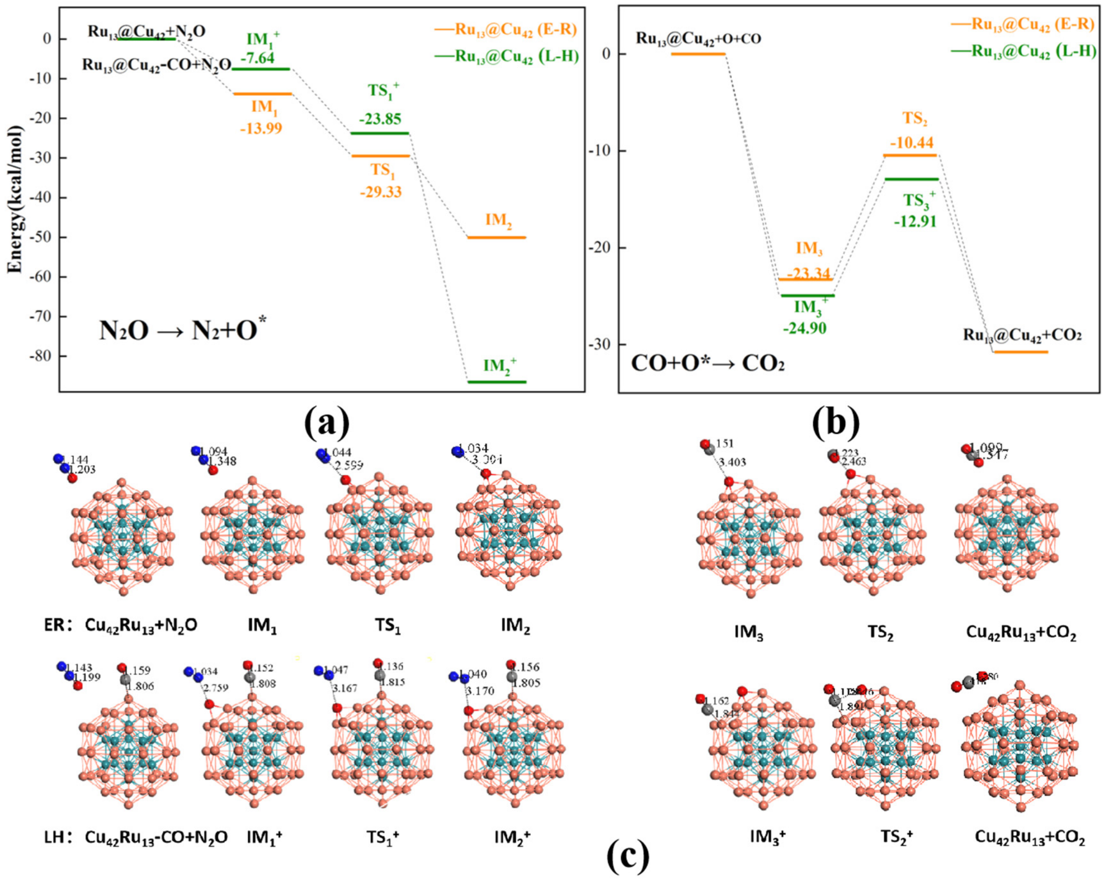

Figure S4. (a) and (b) Potential energy profiles for N_2O decomposition to N_2 on the $Ru_{13}@Cu_{42}$ cluster. (c) Corresponding optimized intermediates and transition states involved in CO oxidation on the $Ru_{13}@Cu_{42}$ cluster.

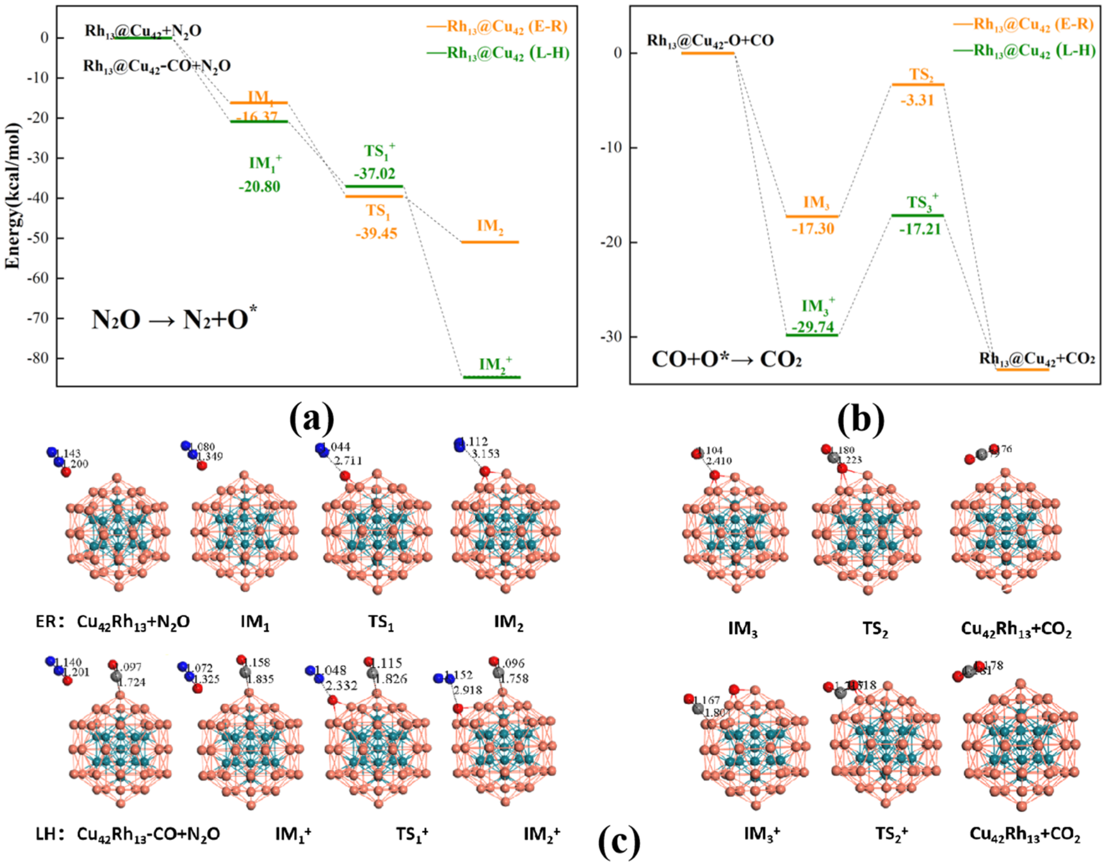

Figure S5. (a) and (b) Potential energy profiles for N_2O decomposition to N_2 on the $Rh_{13}@Cu_{42}$ cluster. (c) Corresponding optimized intermediates and transition states involved in CO oxidation on the $Rh_{13}@Cu_{42}$ cluster.

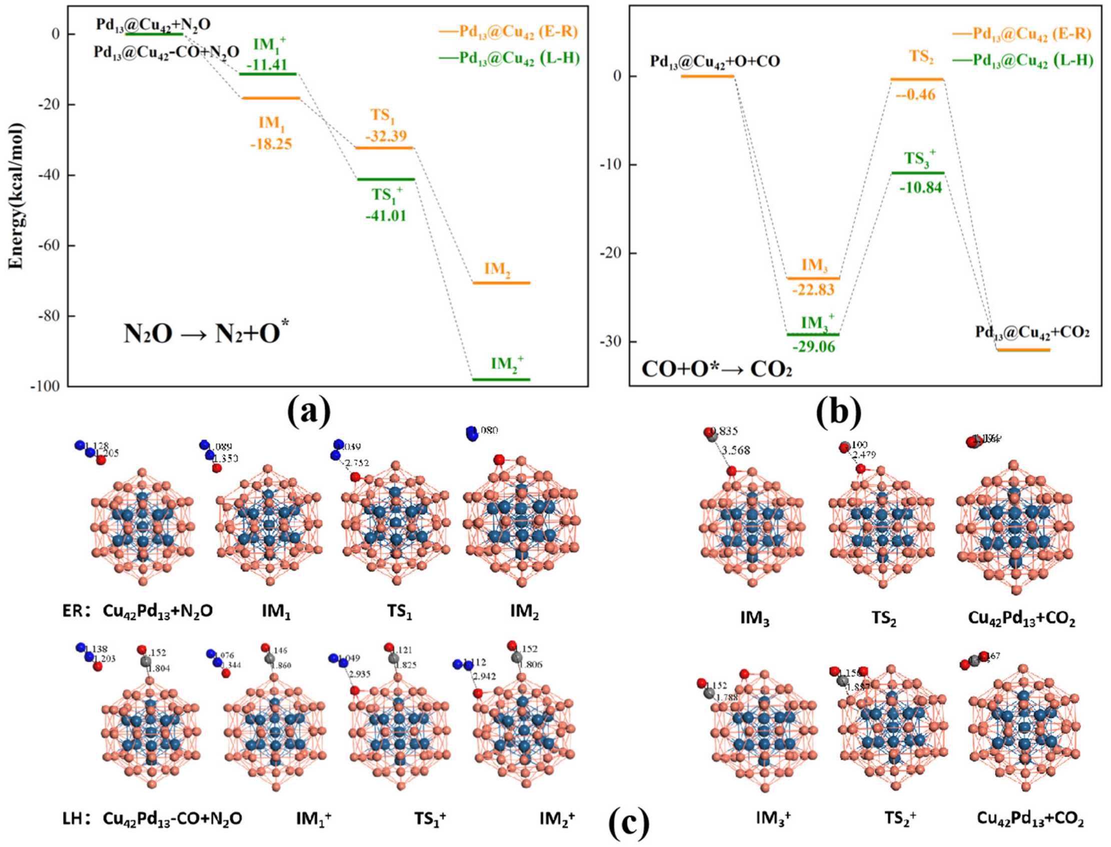

Figure S6. (a) and (b) Potential energy profiles for N_2O decomposition to N_2 on the $Pd_{13}@Cu_{42}$ cluster. (c) Corresponding optimized intermediates and transition states involved in CO oxidation on the $Pd_{13}@Cu_{42}$ cluster.

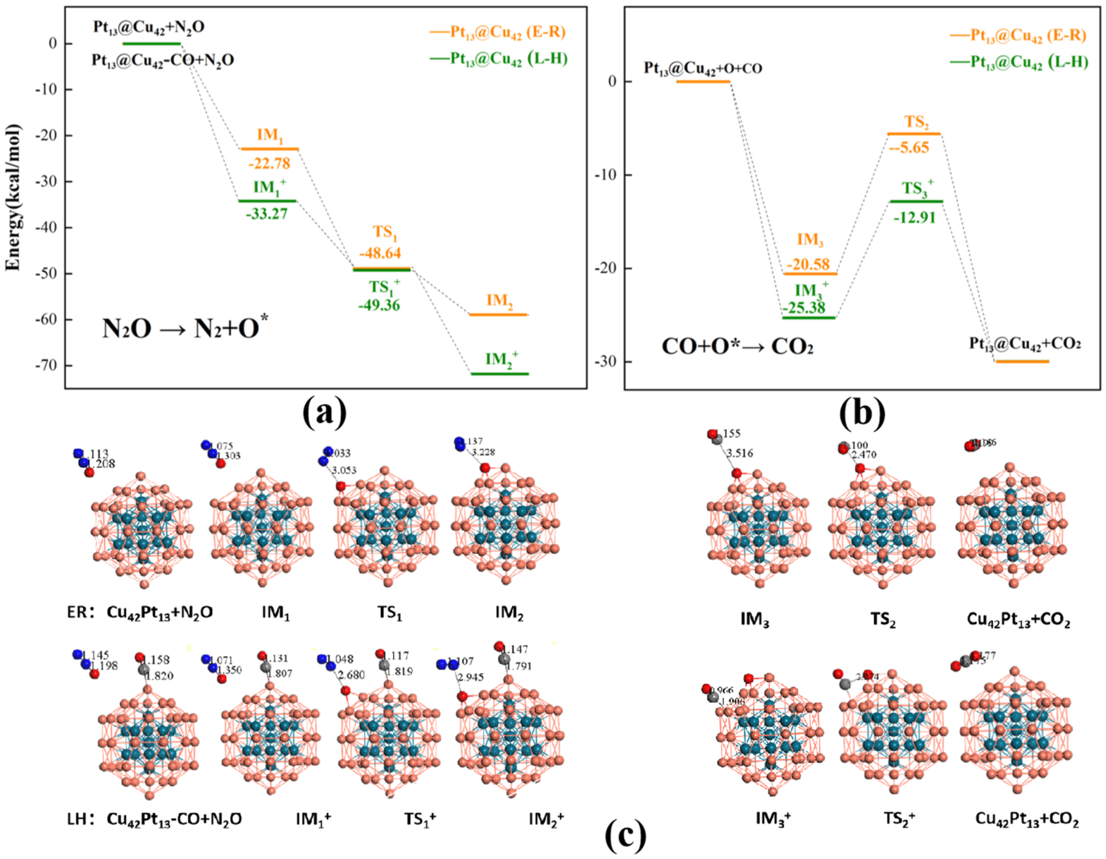

Figure S7. (a) and (b) Potential energy profiles for N_2O decomposition to N_2 on the $Pt_{13}@Cu_{42}$ cluster. (c) Corresponding optimized intermediates and transition states involved in CO oxidation on the $Pt_{13}@Cu_{42}$ cluster.

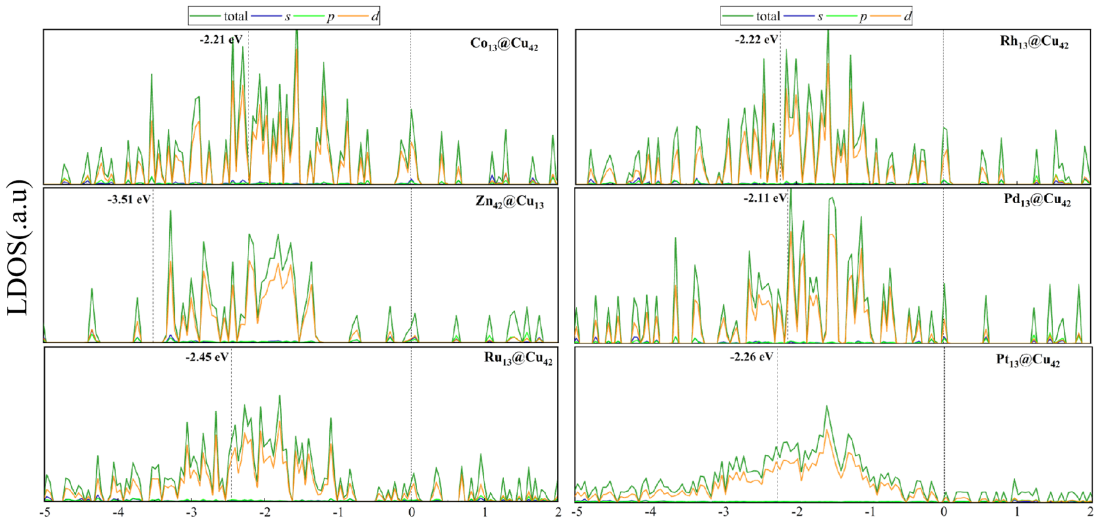

Figure S8. Local density of states (LDOS) of various $M_{13}@Cu_{42}$ clusters (M = Cu, Co, Ni, Zn, Ru, Rh, Pd, Pt). For each system, the HOMO level marked by the black dashed line is shifted to zero, and the red dashed lines and the numbers next to them indicate the d band center.

Table S1. Adsorption energies of different adsorption modes (N, O) and different adsorption sites for N_2O on Cu_{55} cluster (ΔE_{ads}), charge transfer between the molecule and cluster (CT).

	Property	H_1	H_2	B_1	B_2	T_1	T_2
N	ΔE_{ads} (eV)	−7.38	−7.15	−7.61	−7.38	109.77	−7.38
	CT (e)	0.11	0.11	0.11	0.11	0.49	0.11
O	ΔE_{ads} (eV)	−48.43	−53.27	−53.27	−53.27	−3.46	−53.27
	CT (e)	0.96	0.97	0.97	0.97	0.03	0.97

Table S2. The molecular energies, Zero-point energy (ZPE), entropic corrections ($T\Delta S$) to the free energies and (ZPE−TΔS) values of the gas-phase. The Zero-point energy (ZPE) and entropic corrections (TΔS) at T = 298.15 K to the free energies values were taken from NIST-JANAF

thermodynamic table.

	N_2	N_2O	CO	CO_2
E (eV)	−383.49	−493.48	−340.83	−529.23
ΔG (eV)	−16.20	−20.95	−14.24	−22.61
ZPE (ev)	3.46	6.91	3.23	7.15
$T\Delta S$ (eV)	13.37	17.06	15.45	14.99

Table S3. The Gibbs free energies of the adsorbed species at T = 298.15 K. The Zero-point energy (ZPE) and entropic corrections (TΔS).

E-R		Cu	Co	Ni	Zn	Ru	Rh	Pd	Pt
	$\Delta G N_2O$ (eV)	−52.12	−51.19	−47.04	−51.42	−50.96	−51.65	−71.49	−58.80
	$\Delta G CO$ (eV)	−31.13	−32.28	−36.43	−32.05	−32.28	−31.59	−11.99	−24.44
L-H		Cu	Co	Ni	Zn	Ru	Rh	Pd	Pt
	$\Delta G N_2O$ (eV)	−88.32	−93.16	−89.01	−92.70	−102.37	−100.31	−113.46	−90.16
	$\Delta G CO$ (eV)	2.77	7.84	4.38	6.92	4.84	8.30	9.69	4.84
